# Fecal Microbiota Transplantation for Autism Spectrum Disorder in Children: Results from a Prospective Open-Label Controlled Observational Study

**DOI:** 10.3390/medicina62010065

**Published:** 2025-12-28

**Authors:** Dominykas Varnas, Arnas Kunevičius, Aurelijus Burokas, Vaidotas Urbonas

**Affiliations:** 1 Clinic of Children’s Diseases, Faculty of Medicine, Vilnius University, 03101 Vilnius, Lithuania; 2 Department of Biological Models, Institute of Biochemistry, Life Sciences Center, Vilnius University, 10257 Vilnius, Lithuania; arnas.kunevicius@gmc.vu.lt (A.K.);

**Keywords:** autism spectrum disorder, fecal microbiota transplantation, pediatric, FMT, ASD

## Abstract

*Background and Objectives*: Autism spectrum disorder (ASD) is a prevalent neurodevelopmental disorder with an increasing global incidence. Gut microbiota dysbiosis is believed to be playing a role in ASD pathogenesis. Fecal microbiota transplantation (FMT) is emerging as a potential therapeutic strategy to alleviate ASD-related and gastrointestinal symptoms, but data in pediatric ASD populations remain limited. *Materials and Methods*: We conducted a prospective, single-center, open-label controlled study to evaluate the efficacy of colonoscopic FMT in children with ASD. Participants were allocated to two groups: an intervention group that underwent a single FMT procedure and a control group. Gastrointestinal Symptoms Rating Scale (GSRS), Autism Diagnostic Observation Schedule (ADOS), Childhood Autism Rating Scale (CARS), Child Behavior Checklist (CBCL), and Parent Global Impression (PGI-R) scales were assessed for both groups at baseline and at set time points. *Results*: 30 participants were enrolled, with 15 in each group. At 8 weeks, no significant between-group differences were observed for the prespecified primary endpoint, change in ADOS scores. The intervention group showed significantly greater improvements in CARS (*p* < 0.001), PGI-R (*p* < 0.001), CBCL Internalizing Problems (*p* = 0.001), and GSRS (*p* = 0.037) compared with controls; CARS and PGI-R improvements persisted at 6 months. Within the intervention group, sustained improvements were noted in CARS, GSRS, and PGI-R up to 18 months. No serious adverse events were observed; three mild, self-limited adverse events were recorded following FMT. *Conclusions*: Colonoscopic FMT was associated with significant short-term improvements in gastrointestinal and caregiver-reported ASD symptoms (CARS), but not in ADOS scores. Some effects persisted long-term. However, due to a lack of blinding and possible selection bias, these findings should be interpreted as exploratory. Larger randomized controlled trials are needed to confirm efficacy and optimize protocols.

## 1. Introduction

ASD is a neurodevelopmental disorder characterized by persistent deficits in social interaction and communication, along with restricted, repetitive, and inflexible patterns of behavior, interests, or activities that are atypical for the individual’s age and sociocultural context [1]. The diagnosis can usually be established in children as early as 18–24 months of age [2]. It is a prevalent neurodevelopmental disorder with a number of cases estimated at around 28.3 million globally as of 2019 [3]. Over the last two decades, the prevalence of ASD has been increasing steadily from 1 in 150 [4] to 1 in 36 as of 2020 in the USA alone [5]. Predominantly, males are affected more often, with a male-to-female ratio of 3:1 [6]. There is no curative treatment for core ASD symptoms. The main treatment strategies involve behavioral and psychological interventions, while pharmacological options provide only a partial relief to adverse adaptive behaviors and comorbidities [7].

ASD pathogenesis is multifactorial and complex. Hundreds of genes have been identified to play a role in autism through neural development and synaptic function [8]. However, these genes can account for just 10–20% of cases [9]. More than 50% of neurobiological changes could be attributed to non-heritable factors [10]. Environmental triggers [11], immune system dysregulation, neuroinflammation [12], and the gut microbiota dysbiosis [13] are all implicated in ASD pathogenesis. Even though numerous advances have been made in recent years, the precise mechanisms, the interplay of different risk factors, and the influence on neurodevelopment remain incompletely understood [14,15].

Patients with ASD often exhibit a high comorbidity with gastrointestinal symptoms (GIS). GIS are found in about half the children with ASD. This rate is three to four times more than in neurotypical children (NC) [16,17,18]. Children with ASD were approximately six times more likely to experience multiple GIS than typically developing children. Children with ASD and co-occurring GIS exhibit more behavioral challenges—including self-injury, sensory sensitivities, sleep disturbances, attention difficulties, and aggressive behaviors—than those without GIS [19].

The gut microbiota has been proposed to play an important role in ASD [20,21]. Intestinal microbiota composition differs between children with ASD and neurotypical children [22,23,24]. This difference is seen in both human and animal models [25,26,27]. An important pilot study by Sandler et al., though limited in scope, demonstrated a short-term improvement in ASD symptoms in children during treatment with a minimally absorbed oral antibiotic (vancomycin). This study is among the earliest to provide evidence of the potential effectiveness of gut microbiota modulation therapy in children with ASD [28]. While the concept of the gut microbiota and brain connection was longstanding [29], one of the earliest mentions of the term “gut–brain axis” (GBA) is in a 2011 study by Grenham et al., in which the authors explored the complex bidirectional communication network between the central nervous system (CNS) and the gastrointestinal microbiota [30]. Since then, several possible mechanisms underlying the role of the microbiota and GBA in the pathogenesis of ASD have been elucidated [31,32].

The intestinal barrier is a semipermeable barrier and plays a pivotal role in ASD pathogenesis and in human health [33]. Increased intestinal permeability (sometimes referred to as “leaky gut”) allows bacterial products, allergens, and toxins to enter the gut, triggering local inflammation and reaching systemic circulation. De Magistris et al. found that 36.7% of children with ASD exhibited abnormal intestinal permeability, compared to less than 5% of controls [34]. Bacterial components such as lipopolysaccharides (LPS) can trigger low-grade neuroinflammation that can induce changes in the CNS [35]. Healthy gut microbiota maintains intestinal barrier integrity through multiple mechanisms. Beneficial bacteria, such as *Lactobacillus* and *Bifidobacterium*, promote tight junction stability. *Akkermansia muciniphila* enhances mucin production and reinforces the mucus layer, preventing pathogen adhesion to the epithelial surface. Commensal bacteria regulate gut-associated lymphoid tissue, promote regulatory T cell activity, and limit Gram-negative bacterial overgrowth. This helps reduce LPS leakage into circulation [36].

The gut microbiota produces different metabolites, including short-chain fatty acids (SCFAs) [37]. Alterations in the composition of the gut microbiota and its metabolites have been observed in both individuals with ASD and ASD animal models [38,39,40]. Studies reveal that fecal microbiota alterations resulting in an imbalance of certain SCFAs-producing bacteria can lead to exacerbation of ASD symptoms [41,42]. SCFAs modulate the inflammatory status of intestinal epithelial cells and inhibit LPS-induced cytokine production. They also play roles in energy metabolism, influence hormone secretion, and strengthen gut barrier integrity [43,44,45,46,47]. SCFAs act locally but can also cross the blood–brain barrier (BBB) via monocarboxylate transporters [48].

Certain gut bacteria synthesize neurotransmitters or their precursors that influence CNS function [49]. One of them is γ-Aminobutyric acid (GABA), mainly produced by *Lactobacillus* spp. and *Bifidobacterium* spp. [50,51], which is a primary inhibitory neurotransmitter. It controls neuronal excitability, participating in motility, immune cell activity, and secretory gastrointestinal functions [52]. Dysregulation of GABA signaling is a well-documented contributor to the pathophysiology of various neurological disorders, including ASD [53]. Gut microbiota alterations can dysregulate catecholaminergic systems, contributing to behavioral symptoms such as altered reward processing, attention deficits, heightened stress reactivity, and repetitive behaviors [54,55]. Dopamine pathway dysfunction has been implicated in ASD, particularly in relation to social motivation, executive function, and cognitive flexibility [56]. Norepinephrine imbalances are associated with sensory hypersensitivity and autonomic dysfunction. This causes challenges in sensory processing, autonomic regulation, and experiencing heightened responses to sensory input [57]. Additionally, epinephrine’s role in the stress response system suggests that microbial influences on catecholamine production may lead to difficulties in tolerating novel or benign environmental stressors [58]. Serotonin (5-HT) is involved in the regulation of gastrointestinal tract motility and secretion [59]. In the CNS, 5-HT signaling pathways are implicated in regulating mood, sleep, social behavior, cognitive flexibility, and neurodevelopment, including sensory development [60], all of which contribute to the pathophysiology of ASD [61]. While 95% of serotonin is produced by enterochromatophilic cells located within the intestinal mucosa [62], some gut microorganisms (*Escherichia* spp., *Candida* spp., *Enterococcus* spp.) can either produce or, mostly, indirectly influence the metabolism of 5-HT [63,64,65].

Unlike SCFAs, due to the BBB, neurotransmitters are unable to cross into the CNS (with the exception of low quantities of GABA) [66]. Their effects on the CNS are mediated by several mechanisms. Dysbiosis in the gut can alter tryptophan metabolism, reducing its availability as a serotonin precursor in the brain [67]. The vagus nerve provides bidirectional communication between the gut and the brain. Neurotransmitters produced in the gut can activate vagal afferents, which then modulate CNS function. Vagal nerve stimulation has been shown to improve core symptoms of ASD [68,69]. Additionally, a healthy gut microbiota is essential in preserving BBB integrity. SCFAs and their ability to modulate BBB permeability are well-known [70], but other microbial products, namely trimethylamine N-oxide, LPS, and secondary bile acid metabolites, also contribute [71,72]. A Disturbed BBB is not able to effectively prevent translocation of immune cells, inflammatory mediators, and microglial activation, all of which result in increased neuroinflammation, which is an important pathophysiological aspect of ASD [73,74].

Patients with ASD have dysfunction of the hypothalamic–pituitary–adrenal (HPA) axis and the autonomic nervous system, resulting in altered responses to stress and cortisol levels characteristic of ASD [58]. Gut microbiota-related mediators (prostaglandins, cytokines, microbial antigens) activate the HPA axis or suppress it, while the neurotransmitters they produce can, through the vagal nerve, activate the nucleus of the solitary tract and, in turn, the HPA axis [75]. Studies were conducted with germ-free animals that displayed exaggerated HPA axis responses to stress, which were normalized by introducing specific microbial species into their gut [76].

In light of this, microbiota modulation arose as a possible therapy to alter ASD and comorbid symptom severity. Probiotic/prebiotic supplementation has been researched in multiple studies, but current evidence is inconclusive due to a lack of high-quality randomized, double-blind, and placebo-controlled trials. Recommendations cannot be made regarding the efficacy of probiotics/prebiotics in ASD [77,78,79,80]. Given the limitations of probiotics, alternative microbiota-based interventions have gained attention. FMT is an established method for treating *Clostridioides difficile* infection [81] and is being researched for possible therapeutic uses in inflammatory bowel diseases, irritable bowel syndrome, metabolic syndrome, Parkinson’s disease, depression, and other conditions [82]. Unlike conventional probiotics, which are limited to one or several bacteria, FMT contains over a thousand bacterial species [83] as well as native gut viruses, archaea, fungi, and metabolites. A landmark study by Kang et al. showed promising FMT results by reducing autism and GI symptoms in children with ASD [84]. The effect was sustained even at 2 years of follow-up [85]. However, there is still a lack of understanding about the optimal strategy, including delivery route, donor screening, stool preparation, clinical indications, safety, and robust data regarding efficacy [82,86].

We present data from the first prospective, single-center, open-label, controlled study in Europe on the efficacy of FMT in pediatric patients with ASD.

## 2. Materials and Methods

### 2.1. Objective

The objective of this study was to evaluate the effectiveness of the FMT procedure as an investigatory treatment for children with ASD, focusing on its impact on both GIS and ASD symptoms.

### 2.2. Study Design and Ethical Considerations

This open-label study was performed at a tertiary university hospital between 2022 and 2024 and involved two groups of children with ASD aged 3–6 years. A total of 39 children were assessed for eligibility; nine did not participate in the study due to not meeting inclusion criteria (*n* = 4) or declining participation prior to informed consent (*n* = 5), and no study-related data were collected from these patients. The final study population consisted of 15 children in the intervention group who underwent a single FMT (ASDi group) and 15 children in the control group (ASDc), who did not receive FMT.

This study was conducted in accordance with the Declaration of Helsinki. It was approved by the Vilnius Regional Bioethics Committee, Vilnius University. Approval number 2022/4˗1400˗893. Written informed consent was obtained from all parents or legal guardians prior to the enrollment of each participant. Approval date: 12 April 2022.

Participants were not assigned to receive FMT by the research protocol. In our institution, FMT is performed under a pre-existing hospital clinical protocol that operates independently of any research activity. Decisions regarding whether a child should undergo FMT were made exclusively by a multidisciplinary clinical consilium based on individual clinical indications, independent of study.

Parents of children with ASD who were referred to our hospital to explore the possibility of FMT were informed about the procedure and were separately invited to participate in the present observational study. Children who underwent FMT under the clinical protocol and whose parents provided informed consent for study participation were included in the intervention group.

**Figure 1:**
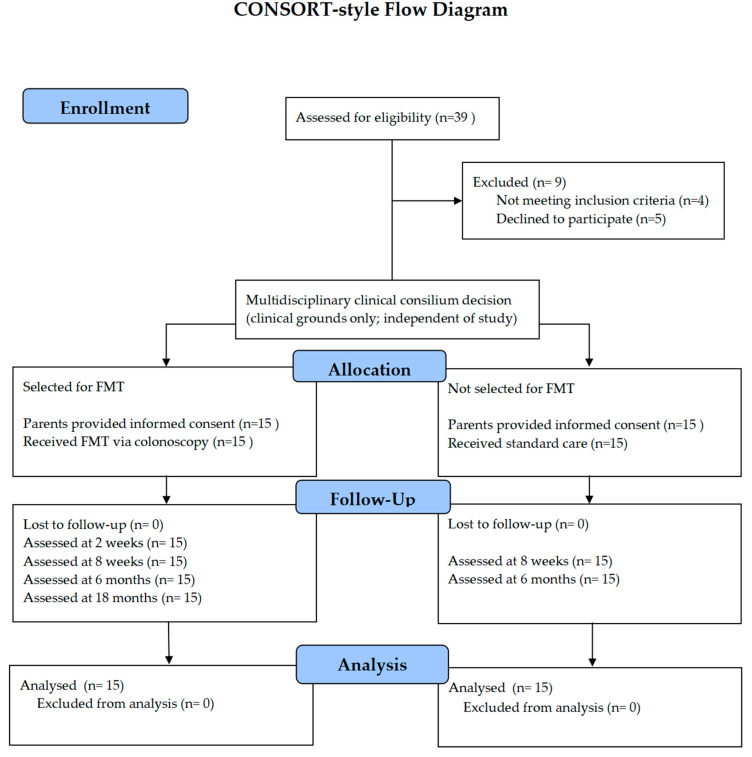
The CONSORT-style flow diagram shows participant flow through study enrollment, clinical allocation, and in-person follow-up assessments. Parents’ decision to decline study participation did not affect clinical care; however, no study-related data were collected from these patients.

The control group consisted of children with ASD receiving standard care at our hospital during the same period. Parents of these children were also invited to participate in the study, but their children did not receive FMT. No randomization or matching was applied (Figure 1).

At our institution, standard care for children with ASD consists of individualized, multidisciplinary non-pharmacological interventions, including kinesitherapy, speech and language therapy (logotherapy), occupational therapy (ergotherapy), structured teaching, and art therapy. The selection, duration, and intensity of these interventions are tailored to each child and determined by treating developmental–behavioral pediatricians. Both groups were advised to continue their usual care throughout the study period. No protocol-mandated restrictions or changes to behavioral or pharmacological treatments were imposed as part of the study, and any therapy or medication decisions during follow-up were made at the discretion of the treating clinicians as part of routine care.

Children whose parents declined participation in the study continued to receive clinically indicated treatment; however, no study-related data were collected from these patients.

Patients were excluded from the study if they used antibiotic within 2 months or probiotic within 1 month prior to the procedure, or patient has been diagnosed inflammatory bowel disease (IBD), infectious gastrointestinal disease within the past 3 months, celiac disease, eosinophilic esophagitis, malnutrition, clinically significant structural brain abnormalities, or a history of abdominal surgery within the past 6 months.

To assess gastrointestinal symptoms, parents completed a modified GSRS [84]. The GSRS is a self-report questionnaire that utilizes a 7-point Likert scale, where responses range from 1 (asymptomatic) to 7 (very severe), with intermediate categories: 2 (slight), 3 (mild), 4 (moderate), 5 (moderate to severe), and 6 (severe).

Assessment of ASD symptoms was performeed using CARS and ADOS. CARS is a 15-item health care professional-rated measure with total scores ranging from 15 to 60, where higher scores indicate greater ASD symptoms [87]. ADOS is a standardized, semi-structured assessment administered by a single trained clinician [88]. The ADOS involves direct observation of social interaction, communication, play, and restricted/repetitive behaviors. To enable comparison across modules and developmental levels, raw scores were converted to calibrated severity scores (normalized ADOS scores), which provide a standardized measure of autism symptom severity.

The PGI-R is a parent-reported instrument designed to capture perceived changes in overall functioning and specific symptom domains in children with ASD [89]. It consists of 11 domains, including social interaction, communication, behavior, daily functioning, and other relevant developmental aspects. Each domain is rated on a 7-point scale of −3 to +3 (−3 = much worse, 0 = no change, and +3 = much better). The final score is the average score of all 11 domains. At baseline, all participants were assigned a PGI-R score of 0, reflecting no perceived change from the child’s usual condition at study entry and serving as the reference anchor for subsequent assessments. The PGI-R was selected because it provides a clinically meaningful, parent-centered assessment of behavioral and functional changes that may not be fully captured by clinician-administered scales.

Finally, behavioral and emotional functioning in children was assessed using the CBCL 1.5–5, part of the ASEBA [90]. It evaluates multiple domains, including internalizing, externalizing, and total behavioral problems, as reported by parents. Each item is rated on a 3-point scale (0 = not true, 1 = somewhat or sometimes true, 2 = very true or often true).

#### 2.2.1. ASD Intervention Group

Before FMT, parents were asked to complete the GSRS and CBCL questionnaires. ASD symptoms were evaluated by a psychologist using the ADOS and CARS during a structured consultation.

After the FMT procedure, the GSRS and PGI-R questionnaires were completed at 1, 2, 3, 4, 8 weeks, and 6 and 18 months post-procedure. The CBCL questionnaire was asked to be completed at 2, 8 weeks, 6 months, and 18 months post-procedure. The ASD symptoms were evaluated using ADOS and CARS at 2, 8 weeks, 6 months, and 18 months post-procedure.

#### 2.2.2. ASD Control Group

At the time of inclusion in the study, parents were asked to complete the GSRS and CBCL questionnaires. A psychologist evaluated ADOS and CARS scores. Follow-up assessments were conducted at 8 weeks and 6 months post-intervention. Parents completed the GSRS, CBCL, and PGI-R questionnaires, and autism symptoms were further evaluated by a psychologist using the ADOS and CARS.

This clinical study was approved by the local Ethics Committee for Scientific Research. Written informed consent was obtained from parents or caregivers before enrollment.

### 2.3. Intervention

For the FMT procedure, patients were prepared with 1.5–3 L polyethylene glycol 4000 (Fortrans) or sodium picosulphate (Picoprep) solution (weight-based dosage) the day before the procedure and in the morning.

No antibiotic pretreatment was administered prior to fecal microbiota transplantation, in accordance with institutional practice and in the absence of standardized pediatric recommendations.

#### 2.3.1. Donor Eligibility and Exclusion Criteria

Fecal microbiota donors were three unrelated healthy children aged 3–7 years. Donors were required to be asymptomatic at the time of donation, and to have normal bowel habits and stool consistency. Donors with a history of food allergy were excluded. Other exclusion criteria included the use of antibiotics, probiotics, or any other medications 30 days prior to donation. Donors with a personal or family history of chronic diseases were excluded. Donor eligibility was assessed using a structured medical interview.

All donor screening investigations were performed within four weeks prior to fecal microbiota transplantation. Donors were screened once before stool donation, and only those with negative results on all screening tests were eligible to participate.

#### 2.3.2. Stool Screening Panel

Stool screening included microscopic examination for protozoa and helminth ova and fecal occult blood testing. Viral testing was performed for rotavirus, norovirus, and adenovirus. Bacteriological (PCR) testing included *Campylobacter* spp., *Salmonella* spp., toxin-producing *Escherichia coli*, *Shigella* spp., *Vibrio* spp., *Yersinia* spp., Listeria monocytogenes, methicillin-resistant Staphylococcus aureus (MRSA), vancomycin-resistant Enterococcus (VRE), and detection of Clostridioides difficile toxins A and B.

#### 2.3.3. Blood Screening Panel

Blood samples were obtained from all donors to exclude transmissible infections. Serological testing included: Hepatitis A virus IgM, Hepatitis B surface antigen (HBsAg), Hepatitis C virus antibodies (anti-HCV), Cytomegalovirus (CMV) IgM, Epstein–Barr virus (EBV) IgM, Human immunodeficiency virus (HIV) testing, Syphilis screening using the rapid plasma reagin (RPR) test.

Additional laboratory testing included: Complete blood count with erythrocyte sedimentation rate (ESR) and C-reactive protein (CRP).

#### 2.3.4. Stool Collection, Handling, and Administration

Stool samples were collected from donors and stored at +4 °C. In all cases, the interval between stool sample collection and FMT was less than 8 h. Stool samples were mixed with 150–200 mL of 0.9% NaCl and manually homogenized. The suspension was filtered to remove particulate matter. After preparation, the fecal suspension was administered within 1 h.

Prepared fecal microbiota suspension (final volume approximately 150–250 mL, depending on stool quantity) was administered during colonoscopy with delivery to the cecum. All procedures were performed under general anesthesia using sevoflurane administered via face mask. The total duration of the fecal microbiota transplantation procedure ranged from 10 to 20 min. No additional antiemetic or antispasmodic medications were administered during the procedure.

### 2.4. Statistical Analysis

Descriptive statistics were calculated for all dependent variables at each assessment time point. Variables are presented as means ± standard deviations (SD) with 95% confidence intervals (CI), while non-normally distributed variables are reported as medians with interquartile ranges (IQR).

All statistical analyses were performed using IBM SPSS Statistics (version 27; IBM Corp., Armonk, NY, USA) and Python (version 3.10; Python Software Foundation, Wilmington, DE, USA) for effect size and correction computations. The primary objective was to evaluate the effects of FMT on behavioral and gastrointestinal symptoms in children with ASD. The primary outcome was the change in normalized ADOS score at 8 weeks post-intervention.

Due to the small sample size (*n* = 15 per group), nonparametric tests were used throughout. Baseline characteristics and outcome scores were compared between the intervention and control groups using the Mann–Whitney U test.

Between-group comparisons at 8 weeks and 6 months were based on change scores from baseline (follow-up minus baseline). For outcomes with significant baseline imbalance (CBCL externalizing and total subscales, GSRS), a rank-based analysis of covariance (Quade test) was applied to adjust for baseline values.

Within-group longitudinal changes in the intervention group were assessed using the Friedman test. If significant, post hoc comparisons were conducted using the Wilcoxon signed-rank test in a predefined hierarchical order: Baseline vs. 2 weeks, followed by 8 weeks, 6 months, and 18 months. Testing stopped at the first non-significant comparison to control the familywise error rate, and no additional corrections (e.g., Bonferroni) were applied. Although all post hoc Wilcoxon comparisons are presented for completeness and transparency, only comparisons up to the first non-significant result within each outcome were considered confirmatory under the hierarchical gatekeeping procedure; subsequent comparisons are reported descriptively and should be interpreted as exploratory.

This gatekeeping procedure was chosen to balance statistical rigor with interpretability. For PGI-R, the default baseline score was 0, and comparisons were made relative to the anchor point.

Effect sizes for Wilcoxon tests were calculated using the formula $\left(r=\frac{Z}{\sqrt{N}}\right)$, where Z is the Wilcoxon test statistic and N is the number of observations (N = 15). Two-sided *p*-values < 0.05 were considered statistically significant.

No a priori sample size or power calculation was performed, as this study was conceived as an exploratory observational investigation conducted within a clinical care framework rather than as a confirmatory randomized trial.

## 3. Results

Baseline Characteristics and Group Comparisons

At baseline, no statistically significant differences were observed between the intervention and control groups on primary measures of autism severity, including normalized ADOS scores (*p* = 0.25) and CARS scores (*p* = 0.367). However, significant differences were noted in GSRS (Median IQR19.0 [17.0–23.0] vs. 27.0 [23.0–38.0]), CBCL Externalizing (10.0 [7.0–20.0] vs. 23.0 [11.0–27.0]) and CBCL Total scores (31.0 [27.0–50.0] vs. 49.0 [40.0–58.0]), with the intervention group reporting more severe symptoms at baseline (Table 1).

Descriptive statistics for both intervention and control groups can be found in Appendix A (Table A1 and Table A2, respectively).

Between-Group Differences at 8 Weeks and 6 Months

Mann–Whitney U tests of change scores revealed that, at 8 weeks post-intervention, the intervention group demonstrated significantly greater improvements in CARS by (*p* < 0.001), PGI-R (*p* < 0.001), and CBCL Internalizing Problems (*p* = 0.001) scores compared to the ASDc group. These effects were sustained at 6 months for CARS (*p* = 0.045) and PGI-R (*p* = 0.007), but not for CBCL Internalizing Problems (*p*= 0.25). No statistically significant group differences were observed in ADOS score at both time points (*p* = 0.061 and *p* = 0.367) (Table 2). Although no statistically significant between-group differences were observed for ADOS change scores, the corresponding effect sizes were moderate at 8 weeks (r ≈ 0.44) and small-to-moderate at 6 months (r ≈ 0.26), indicating that the non-significant findings may reflect limited statistical power rather than the absence of a clinically meaningful effect.

**Figure 2:**
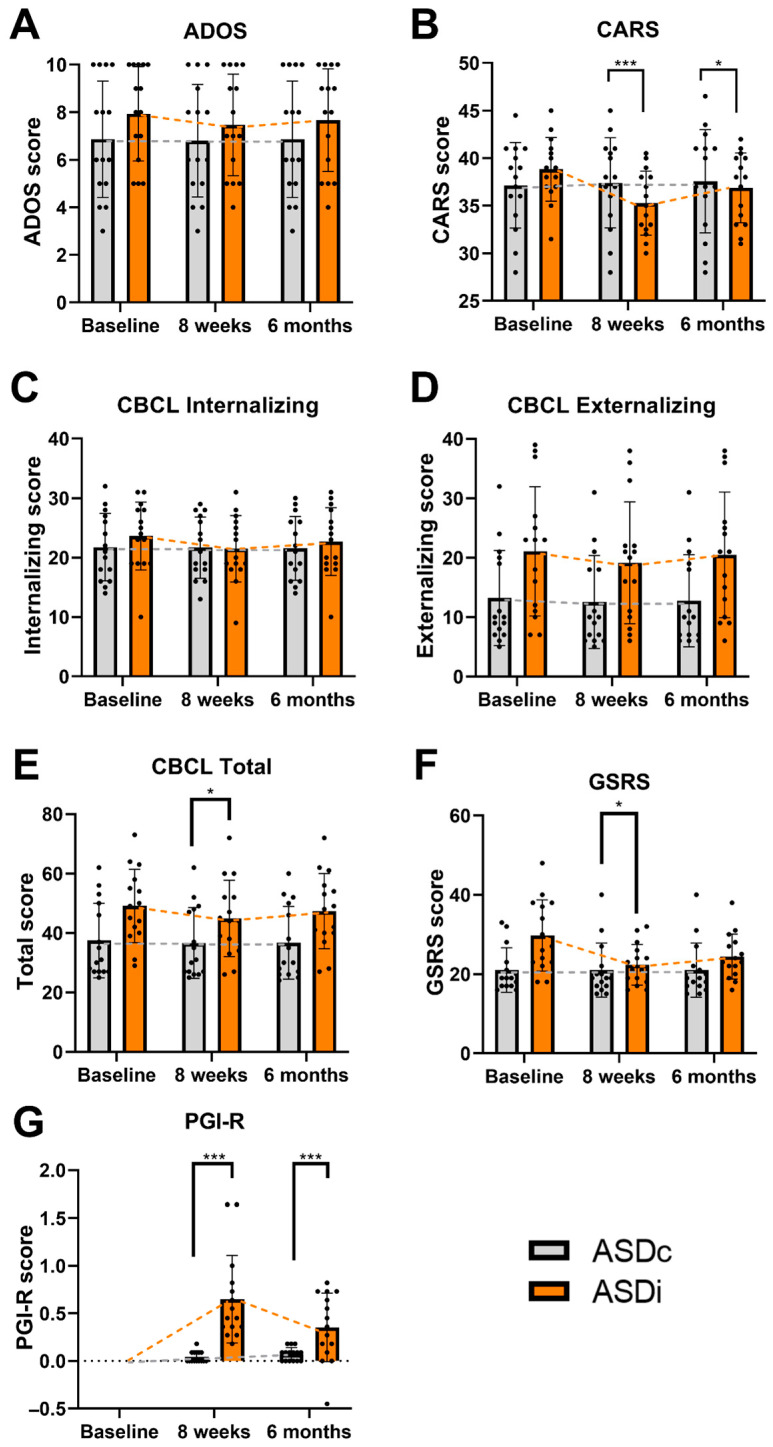
Between-group comparison between the ASDi and control (ASDc) groups at baseline, 8 weeks, and 6 months. (**A**–**G**) Panels depict scores in ADOS, CARS, CBCL subscales (Internalizing, Externalizing, and Total Problems), GSRS, and PGI-R, respectively. The ASDi group demonstrated significantly greater improvement in CARS, PGI-R, GSRS, CBCL Internalizing, and CBCL Total scores at 8 weeks compared with controls. Improvement was sustained at 6 months in CARS, CBCL Internalizing, and PGI-R scores. Data are presented as mean ± SD. * *p* ≤ 0.05, *** *p* ≤ 0.001.

Due to baseline differences, rank-based ANCOVA (Quade test) was performed. After adjustment, the CBCL total problems subscale and GSRS showed significant between-group differences at 8 weeks (*p* = 0.001 and *p* = 0.037, respectively), but the effect was not observed at 6 months. No significant difference was observed on CBCL Externalizing Problems at both time points (Table 3, Figure 2).

Longitudinal symptom changes within the ASDi group

**Figure 3:**
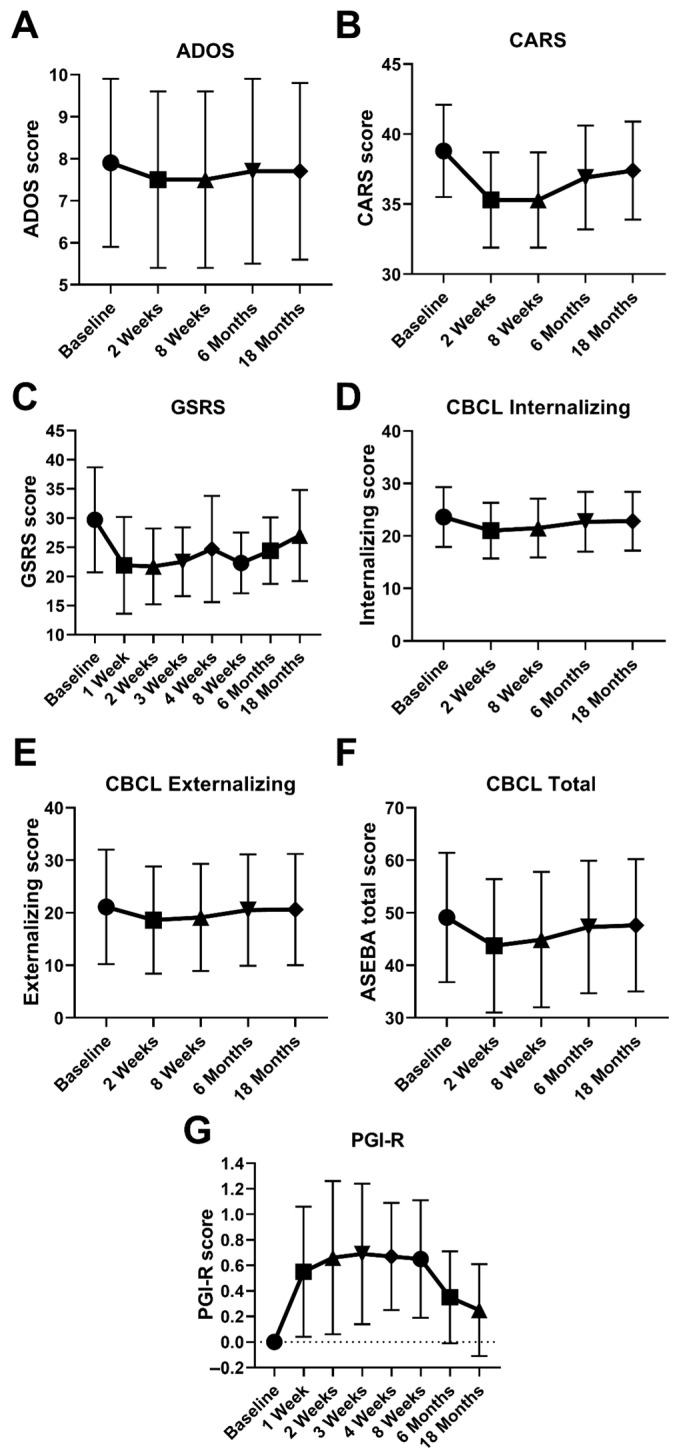
ASDi group symptom scores after FMT. Panels (**A**–**G**) show trajectories of ADOS, CARS, GSRS, PGI-R, and CBCL subscales (Internalizing, Externalizing, and Total Problems) across baseline, 2 weeks, 8 weeks, 6 months, and 18 months. GSRS and PGI-R have additional timepoints at 1, 3, and 4 weeks. Significant reductions were observed in CARS, GSRS, and CBCL Internalizing scores, with sustained improvement in PGI-R ratings throughout follow-up (Wilcoxon signed-rank test *p* < 0.01). Data are presented as median (interquartile range).

Friedman tests showed significant changes over time across all outcomes in the ASDi group (Table 4). Post hoc Wilcoxon signed-rank tests were then applied in a hierarchical fashion to compare baseline scores with each follow-up time point (2 weeks, 8 weeks, 6 months, and 18 months) for all outcome measures (Table 5, Figure 3). Reported are Z statistics, *p*-values, and effect sizes (r). Significant improvements were observed in CARS, GSRS, PGI-R, and CBCL subscales, with varying persistence across time points.

ADOS scores significantly improved at 2 weeks (*p* = 0.031) and 8 weeks (*p* = 0.016), but there were no significant changes at 6 or 18 months. CARS scores showed sustained improvement across all time points (*p* < 0.01).

CBCL internalizing and total problems improved consistently through 18 months. CBCL externalizing scores showed significant early reductions at 2 and 8 weeks (*p* = 0.001), but these gains were not sustained at 6 or 18 months.

GSRS scores demonstrated significant reductions in gastrointestinal symptoms from baseline to all post-intervention time points.

PGI-R scores improved significantly at all follow-up visits compared with baseline (2 weeks: Z = −3.11, *p* = 0.001, r = 0.80; 8 weeks: Z = −4.02, *p* < 0.001; 6 months: Z = −3.62, *p* < 0.001, r = 0.94; 18 months: Z = −2.68, *p* = 0.004, r = 0.69). Improvement was observed in 14 of 15 participants, resulting in extremely large effect sizes. At 8 weeks, the effect size calculation exceeded 1.0 due to the ceiling effect rather than error.

### Safety Outcomes

Three adverse events were recorded following the FMT. Two patients experienced transient diarrhea accompanied by mild abdominal discomfort on the day of the procedure. Symptoms resolved spontaneously on the same day without intervention. One patient developed subfebrile fever and pharyngitis symptoms three days after the procedure, which resolved in an ambulatory setting without treatment and was considered unlikely to be related to FMT. No serious adverse events, procedural complications, or hospitalizations occurred. No unexpected clinical findings were observed during the 18-month follow-up period.

## 4. Discussion

Several previous open-label studies suggested that FMT can improve both gastrointestinal and ASD-related symptoms [84,91,92,93,94,95,96]. However, these studies lacked a control group. Since no standardized intervention model for FMT in children with ASD exists, patient preparation procedures (antibiotics, bowel cleansing, gastric acid suppression), administration routes of FMT, and donor selection varied significantly.

We decided not to use antibiotic pretreatment because the role of antibiotics in facilitating FMT engraftment remains uncertain, with conflicting evidence and no consensus regarding optimal regimens, particularly in pediatric populations with ASD [81,97].

To the best of our knowledge, only two randomized controlled trials (RCTs) have been published with conflicting results. A study by Wang et al. [98] reported improvement in both GI and ASD symptoms, while Wan et al. [99] found no significant ASD-related symptom improvement.

These discrepancies may be explained by differences in study design and patient selection. Wang et al. employed a multi-step intervention, including antibiotic pretreatment and bowel cleansing before repeated oral FMT capsule administration, and reported consistent improvements in both gastrointestinal and ASD symptoms [98]. In contrast, Wan et al. administered two short courses of oral FMT capsules without antibiotics and did not observe significant differences from placebo on primary (SRS-2) or secondary (Vineland-3, ABC) outcomes, despite within-group improvements in both arms [99]. Unlike in Wang and Wan studies, we used one-time FMT via colonoscopy, because it ensures transplant administration to the cecum, and the effectiveness in treating *C. difficile* colitis of this route is comparable to capsules and superior to other administration routes [100]. Importantly, we also used the CARS as one of our outcomes, consistent with Wang et al., which facilitates comparison between studies. In addition, we included the ADOS, a gold-standard, clinician-administered measure that was not applied in any of the mentioned trials. This allowed us to capture both caregiver-reported improvements and clinician-rated outcomes, revealing a divergence that may reflect expectancy bias or differences in sensitivity between instruments.

The primary endpoint, change in ADOS scores at 8 weeks, did not differ significantly between groups. However, several secondary outcomes showed meaningful improvement in the intervention group compared with untreated controls. Children receiving FMT demonstrated greater reductions in CARS scores at both 8 weeks and 6 months, alongside significant improvements in caregiver-reported PGI-R scores. GSRS and CBCL Internalizing and Total Problems improved at the 8-week time point only. Improvements evident at the 8-week time point but not maintained at 6 months may reflect transient changes in gut microbiota after FMT, with partial reversion over time in the absence of repeated dosing. Alternatively, expectancy effects in an open-label design may be strongest shortly after treatment but attenuate during longer follow-up.

The decision to administer a single colonoscopic FMT was driven by practical and methodological considerations. At present, there are no consensus recommendations regarding the optimal number or frequency of FMT procedures for children with ASD [82,86]. Colonoscopic delivery requires general anesthesia and carries a procedural burden, making repeated administrations less feasible, especially in pediatric populations. A single FMT allowed assessment of the clinical impact of a single, clearly defined intervention.

While no differences were detected in ADOS scores between groups, CARS scores showed significant improvements following FMT. Both scales measure autism severity, yet they differ in sensitivity and context. ADOS is examiner-administered, based on structured tasks, which may not fully reflect improvements in daily behavior. ADOS was primarily developed as a diagnostic instrument rather than an outcome measure, and its standardized, time-limited observational format may limit sensitivity to short-term behavioral change [101].

Differences in the psychometric properties of these instruments may also contribute. ADOS has relatively coarse scoring ranges (items scored 0–3, then summed), and total scores are mapped to calibrated severity scores. This compresses variability and may reduce sensitivity to small treatment-related shifts. Previous works noted that ADOS total and calibrated severity scores tend to remain relatively stable over short follow-up intervals, which may further constrain their ability to capture subtle treatment-related improvements over weeks to a few months [102,103,104].

In contrast, CARS includes 15 items scored on a 7-point scale, offering greater resolution to detect subtle improvements. Additionally, CARS incorporates caregiver interview data alongside direct observation, allowing ratings to reflect behavior across a broader range of daily situations like emotional responses and adaptability that may have been influenced not only by changes in core ASD symptoms, but also by improvements in emotional regulation, adaptability, and reduction of GI-related distress [101]. However, CARS is more subjective and may be influenced by expectancy bias, particularly in an open-label design where caregivers were aware of treatment assignment. Placebo responses are well documented in autism intervention studies, with larger placebo effects consistently observed when outcomes are reported by caregivers rather than clinicians [104]. Such expectancy-related effects may preferentially influence global or context-dependent measures such as CARS, whereas structured clinician-administered instruments, such as ADOS, conducted in standardized settings, may be less sensitive to these influences [105]. This difference between scales may partly explain why CARS, but not ADOS, showed significance between groups.

Beyond differences in psychometric properties and possible expectancy effects, the discrepancy between caregiver-reported improvements (CARS, PGI-R) and the lack of significant between-group effects on ADOS may also be viewed in relation to sensory regulation and neurophysiological stress in ASD. Children with ASD and co-occurring sensory processing difficulties often show heightened stress responses. Reductions in physical or sensory discomfort are more often noticed by caregivers in everyday contexts than by clinicians using structured observational tools. It is relevant since improvements in gastrointestinal symptoms following FMT may plausibly reduce ongoing visceral discomfort and associated stress. In turn, parents may observe changes in emotional regulation, adaptability, and daily functioning that are less readily captured by ADOS scores, particularly over relatively short follow-up periods. Although neurophysiological stress markers were not assessed in the present study, this framework offers a plausible mechanistic explanation for the greater sensitivity of parent-based instruments [106].

These considerations indicate that the discrepancy between ADOS and CARS reflects a divergence that may arise from differences in instrument sensitivity, expectancy bias, and measurement context, or all of these, rather than necessarily indicating inconsistency in the observed clinical response.

Analyses within the ASDi group revealed sustained improvements in multiple domains, with CARS, GSRS, and PGI-R persisting up to 18 months post-treatment. However, the control group was followed for only 6 months. Due to this, these long-term findings cannot be attributed solely to the intervention. Long-term developmental trajectories in ASD are highly heterogeneous and influenced by baseline severity, comorbidities, and concurrent interventions, as demonstrated in previous longitudinal studies [107,108]. In addition, although a hierarchical gatekeeping procedure was applied to control the familywise error rate within longitudinal analyses, the evaluation of multiple secondary outcomes across several domains and time points increases the potential for residual Type I error, further supporting an exploratory interpretation of these findings.

These observations support the long-term benefits reported in the follow-up study by Kang et al. [85]. There are no other studies with a long follow-up. Since within-group comparisons cannot fully rule out the placebo effect or natural disease development, these results should be interpreted cautiously.

Finally, we chose the colonoscopic delivery route for FMT as oral routes require either a tube placement or swallowing a significant amount of capsules for several days, which is problematic for children with ASD. The only study comparing the effectiveness of FMT in children with ASD was by Li et al. [91], in which no significant difference was observed between oral or colonoscopic FMT administration.

### Limitations

This study has several methodological limitations. Although outcomes were assessed prospectively, assignment to FMT or standard care was not randomized and followed routine clinical decision-making under established hospital protocol, with parental agreement. Therefore, selection bias remains a possibility.

Baseline differences between groups should also be considered when interpreting the results. The intervention group showed higher GSRS and CBCL scores at the beginning of the study, raising the possibility that some of the observed improvement may partly reflect regression to the mean rather than a true treatment effect. We addressed this by applying baseline-adjusted rank-based ANCOVA (Quade test) for outcomes with significant baseline differences. Nevertheless, statistical adjustment cannot fully eliminate regression to the mean in a non-randomized study.

Follow-up frequency differed between the intervention and control groups. Patients who underwent FMT were monitored more frequently, particularly early after the procedure, reflecting clinical safety considerations given the invasive nature of the intervention. The control group received standard care, and intensive short-term monitoring was not required, thereby avoiding unnecessary burden. This asymmetry in follow-up schedules may have introduced observation bias, particularly for caregiver-reported outcomes, and should be considered when interpreting the results.

No microbiome sequencing or biomarker analyses were performed in this study; therefore, mechanistic interpretations regarding gut–brain pathways remain speculative and cannot be directly inferred from the present data.

While a comparison group was included, the absence of randomization and blinding limits causal inference and increases susceptibility to expectancy bias. The absence of blinding is particularly relevant for caregiver-reported outcomes such as CARS, PGI-R, GSRS, and CBCL, which may overestimate treatment effects. In addition, the small, clinically heterogeneous study population limits generalizability. No a priori power calculation was performed, and with only 15 participants per group, the study was likely underpowered to detect small-to-moderate between-group differences, particularly for clinician-administered measures such as ADOS. Accordingly, the findings should be interpreted as exploratory and hypothesis-generating, underscoring the need for larger, randomized, and blinded trials.

## 5. Conclusions

In this open-label controlled study, the prespecified primary endpoint—change in clinician-administered ADOS scores at 8 weeks—was not significantly different between children who underwent FMT and controls. Secondary outcomes, including gastrointestinal symptoms and caregiver-reported measures of autism symptom severity (CARS), showed improvements in the intervention group. While the persistence of improvements in some domains up to 18 months is encouraging, the absence of randomization and blinding underscores the need for cautious interpretation. Taken together, these findings should be regarded as exploratory and hypothesis-generating, supporting the rationale for larger, blinded, and methodologically rigorous trials with standardized protocols to determine efficacy and the best routes and FMT preparations.

## Figures and Tables

**Table 1:** The comparison of baseline characteristics of participants between study groups.

Measure	Control Group Mean (SD)	Intervention Group Mean (SD)	Median [IQR] Control vs. Intervention	*p*
Age (years)	4.40 (0.83)	5.13 (1.36)	4.0 [4.0–5.0] vs. 5.0 [4.0–6.0]	0.116
ADOS	6.87 (2.45)	7.93 (1.98)	6.0 [5.0–10.0] vs. 8.0 [6.0–10.0]	0.250
CARS	37.13 (4.50)	38.83 (3.34)	38.0 [34.0–41.0] vs. 39.0 [37.0–41.0]	0.367
GSRS	21.00 (5.59)	29.73 (8.97)	19.0 [17.0–23.0] vs. 27.0 [23.0–38.0]	0.002
CBCL Internalizing	21.73 (5.68)	23.60 (5.71)	21.0 [16.0–27.0] vs. 24.0 [19.0–28.0]	0.285
CBCL Externalizing	13.20 (7.99)	21.07 (10.89)	10.0 [7.0–20.0] vs. 23.0 [11.0–27.0]	0.026
CBCL Total Problems	37.40 (12.55)	49.07 (12.35)	31.0 [27.0–50.0] vs. 49.0 [40.0–58.0]	0.015

Data are presented as mean (SD) and median [IQR]. *p*-values were obtained using the Mann–Whitney U test.

**Table 2:** Between-group comparison of change scores (Mann–Whitney U) at 8-week and 6-month time points.

Measure	Time Point	Median (ASDc)	IQR (ASDc)	Median (ASDi)	IQR (ASDi)	*p* (MWU)
ADOS	8 weeks	0.0	[−1.00–1.00]	−1.0	[−2.00–0.00]	0.061
ADOS	6 months	−0.5	[−1.00–0.00]	−1.0	[−3.00–0.00]	0.367
CARS	8 weeks	0.0	[−1.00–1.00]	−4.0	[−5.00–3.00]	**<0.001**
CARS	6 months	−1.0	[−2.00–0.00]	−5.0	[−7.00–3.00]	**0.045**
PGI-R	8 weeks	1.0	[1.00–2.00]	3.0	[3.00–4.00]	**<0.001**
PGI-R	6 months	2.0	[1.00–3.00]	3.0	[3.00–4.00]	**0.007**
CBCL Internalizing	8 weeks	0.0	[−2.00–2.00]	−4.0	[−5.00–2.00]	**0.001**
CBCL Internalizing	6 months	−3.0	[−4.00–2.00]	−4.0	[−6.00–1.00]	0.250

Data are presented as median [interquartile range]. Between-group comparisons were performed using the Mann–Whitney U test. Bold values indicate statistically significant results (*p* < 0.05).

**Table 3:** Between-group comparison (Quade Rank ANCOVA, Baseline-Adjusted Outcomes) of change scores at 8-week and 6-month time points.

Measure	Time Point	Adjusted *p*	Partial Eta^2^
GSRS	8 weeks	**0.037**	0.152
GSRS	6 months	0.240	0.051
CBCL Externalizing	8 weeks	0.309	0.038
CBCL Externalizing	6 months	0.947	0.000
CBCL Total	8 weeks	**0.001**	0.323
CBCL Total	6 months	0.402	0.026

Bold values indicate statistically significant results (*p* < 0.05).

**Table 4:** Over time ASDi group symptoms (Friedman test).

Measure	Total Time Points	Friedman χ^2^	df	*p*-Value
ADOS	5	17.647	4	**0.0014**
CARS	5	33.224	4	**<0.0001**
CBCL Internalizing	5	37.82	4	**<0.0001**
CBCL Externalizing	5	25.192	4	**<0.0001**
CBCL Total Problems	5	34.756	4	**<0.0001**
GSRS	7	28.858	6	**<0.0001**
PGI-R	7	22.484	6	**0.001**

Friedman test used to assess within-group changes across time points in the ASDi group (Baseline, 2 weeks, 8 weeks, 6 months, and 18 months). χ^2^—Chi-square statistic; df—degrees of freedom. Bold values indicate statistically significant results (*p* < 0.05).

**Table 5:** ASDi group Wilcoxon hierarchical post hoc tests.

Outcome	Comparison	Z	*p*-Value	Effect Size (r)
ADOS	Baseline vs. 2 w	−2.449	**0.031**	0.632
ADOS	Baseline vs. 8 w	−2.646	**0.016**	0.683
ADOS	Baseline vs. 6 m	−2	0.125	0.516
ADOS	Baseline vs. 18 m	−1.732	0.25	0.447
CARS	Baseline vs. 2 w	−3.192	**<0.001**	0.824
CARS	Baseline vs. 8 w	−3.208	**<0.001**	0.828
CARS	Baseline vs. 6 m	−2.558	**0.008**	0.66
CARS	Baseline vs. 18 m	−2.453	**0.012**	0.633
CBCL Internalizing	Baseline vs. 2 w	−3.319	**<0.001**	0.857
CBCL Internalizing	Baseline vs. 8 w	−3.078	**<0.001**	0.795
CBCL Internalizing	Baseline vs. 6 m	−2.585	**0.008**	0.667
CBCL Internalizing	Baseline vs. 18 m	−2.489	**0.016**	0.643
CBCL Externalizing	Baseline vs. 2 w	−3.052	**0.001**	0.788
CBCL Externalizing	Baseline vs. 8 w	−3.09	**0.001**	0.798
CBCL Externalizing	Baseline vs. 6 m	−1.83	0.093	0.473
CBCL Externalizing	Baseline vs. 18 m	−1.615	0.143	0.417
CBCL Total	Baseline vs. 2 w	−3.301	**<0.001**	0.852
CBCL Total	Baseline vs. 8 w	−3.31	**<0.001**	0.855
CBCL Total	Baseline vs. 6 m	−2.912	**0.003**	0.752
CBCL Total	Baseline vs. 18 m	−2.503	**0.011**	0.646
GSRS	Baseline vs. 2 w	−3.413	**<0.001**	0.881
GSRS	Baseline vs. 8 w	−3.306	**<0.001**	0.854
GSRS	Baseline vs. 6 m	−2.882	**0.003**	0.744
GSRS	Baseline vs. 18 m	−2.668	**0.007**	0.689
PGI-R	Baseline vs. 2 w	−3.105	**0.001**	0.802
PGI-R	Baseline vs. 8 w	−4.023	**<0.001**	1.039
PGI-R	Baseline vs. 6 m	−3.623	**<0.001**	0.935
PGI-R	Baseline vs. 18 m	−2.677	**0.004**	0.691

Post hoc Wilcoxon signed-rank tests were conducted hierarchically within the ASDi group to compare baseline scores with follow-up time points (2 weeks, 8 weeks, 6 months, 18 months). Significant results are shown in bold. All post hoc comparisons are reported for transparency. In accordance with the predefined hierarchical gatekeeping strategy, only comparisons occurring prior to the first non-significant result were considered confirmatory; subsequent comparisons are exploratory and reported descriptively. Bold values indicate statistically significant results (*p* < 0.05).

## Data Availability

The raw de-identified data presented in this study are available on reasonable request from the corresponding author. The data are not publicly available due to ethical restrictions related to pediatric patient confidentiality.

## Abbreviations

The following abbreviations are used in this manuscript:

ASD	Autism spectrum disorder
FMT	Fecal microbiota transplantation
GSRS	Gastrointestinal Symptoms Rating Scale
ADOS	Autism Diagnostic Observation Schedule
CARS	Childhood Autism Rating Scale
ASEBA	Achenbach System of Empirically Based Assessment
PGI-R	Parent Global Impression
GIS	Gastrointestinal symptoms
NC	Neurotypical children
GBA	Gut–brain axis
CNS	Central nervous system
LPS	Lipopolysaccharides
SCFAs	Short-chain fatty acids
BBB	Blood–brain barrier
GABA	γ-Aminobutyric acid
5-HT	Serotonin
HPA	Hypothalamic–pituitary–adrenal
ASDi	Autism spectrum disorder intervention
ASDc	Autism spectrum disorder control
IBD	Inflammatory bowel disease
RCT	Randomized controlled trial

## Appendix A

**Table A1:** Descriptive statistics, intervention group (ASDi).

Group	Variable	Time Point	Mean ± SD	Median [IQR]
ASDi	ADOS	Baseline	7.9 ± 2.0	8.0 [6.0–10.0]
ASDi	CARS	Baseline	38.8 ± 3.3	39.0 [37.0–41.0]
ASDi	GSRS	Baseline	29.7 ± 9.0	27.0 [23.0–38.0]
ASDi	CBCL Internalizing Problems	Baseline	23.6 ± 5.7	24.0 [19.0–28.0]
ASDi	CBCL Externalizing Problems	Baseline	21.1 ± 10.9	23.0 [11.0–27.0]
ASDi	CBCL Total Problems	Baseline	49.1 ± 12.3	49.0 [40.0–58.0]
ASDi	GSRS	1 week	21.9 ± 8.3	19.0 [17.0–26.0]
ASDi	GSRS	2 weeks	21.7 ± 6.5	19.0 [17.0–23.0]
ASDi	CBCL Internalizing Problems	2 weeks	21.0 ± 5.3	22.0 [18.0–24.0]
ASDi	CBCL Externalizing Problems	2 weeks	18.6 ± 10.2	17.0 [10.0–22.0]
ASDi	CBCL Total Problems	2 weeks	43.7 ± 12.7	43.0 [34.0–49.0]
ASDi	ADOS	2 weeks	7.5 ± 2.1	8.0 [5.0–10.0]
ASDi	CARS	2 weeks	35.3 ± 3.4	36.0 [32.0–38.0]
ASDi	GSRS	3 weeks	22.5 ± 5.9	21.0 [17.0–25.0]
ASDi	GSRS	4 weeks	24.7 ± 9.1	21.0 [18.0–29.0]
ASDi	GSRS	8 weeks	22.3 ± 5.2	21.0 [18.0–26.0]
ASDi	CBCL Internalizing Problems	8 weeks	21.5 ± 5.6	20.0 [18.0–26.0]
ASDi	CBCL Externalizing Problems	8 weeks	19.1 ± 10.2	20.0 [10.0–22.0]
ASDi	CBCL Total Problems	8 weeks	44.9 ± 12.9	44.0 [34.0–52.0]
ASDi	ADOS	8 weeks	7.5 ± 2.1	7.0 [5.0–10.0]
ASDi	CARS	8 weeks	35.3 ± 3.4	35.0 [32.5–38.0]
ASDi	GSRS	6 months	24.4 ± 5.7	23.0 [20.0–28.0]
ASDi	CBCL Internalizing Problems	6 months	22.7 ± 5.7	23.0 [19.0–28.0]
ASDi	CBCL Externalizing Problems	6 months	20.5 ± 10.6	21.0 [10.0–26.0]
ASDi	CBCL Total Problems	6 months	47.3 ± 12.6	48.0 [40.0–56.0]
ASDi	ADOS	6 months	7.7 ± 2.2	8.0 [5.0–10.0]
ASDi	CARS	6 months	36.9 ± 3.7	38.0 [33.0–40.0]
ASDi	GSRS	18 months	27.0 ± 7.8	25.0 [20.0–35.0]
ASDi	CBCL Internalizing Problems	18 months	22.8 ± 5.6	23.0 [19.0–27.0]
ASDi	CBCL Externalizing Problems	18 months	20.6 ± 10.6	21.0 [11.0–28.0]
ASDi	CBCL Total problems	18 months	47.6 ± 12.6	48.0 [41.0–55.0]
ASDi	ADOS	18 months	7.7 ± 2.1	8.0 [6.0–10.0]
ASDi	CARS	18 months	37.4 ± 3.5	37.0 [34.0–41.0]
ASDi	PGI-R	1 week	0.55 ± 0.51	0.45 [0.27–0.91]
ASDi	PGI-R	2 weeks	0.66 ± 0.6	0.45 [0.27–0.91]
ASDi	PGI-R	3 weeks	0.69 ± 0.55	0.55 [0.27–0.91]
ASDi	PGI-R	4 weeks	0.67 ± 0.42	0.64 [0.27–0.82]
ASDi	PGI-R	8 weeks	0.65 ± 0.46	0.45 [0.36–0.82]
ASDi	PGI-R	6 months	0.35 ± 0.36	0.36 [0.09–0.73]
ASDi	PGI-R	18 months	0.25 ± 0.36	0.18 [0.00–0.27]

**Table A2:** Descriptive statistics control group (ASDc).

Group	Variable	Time Point	Mean ± SD	Median [IQR]
ASDc	ADOS	Baseline	6.9 ± 2.4	6.0 [5.0–10.0]
ASDc	CARS	Baseline	37.1 ± 4.5	38.0 [34.0–41.0]
ASDc	GSRS	Baseline	21.0 ± 5.6	19.0 [17.0–23.0]
ASDc	CBCL Internalizing Problems	Baseline	21.7 ± 5.7	21.0 [16.0–27.0]
ASDc	CBCL Externalizing Problems	Baseline	13.2 ± 8.0	10.0 [7.0–20.0]
ASDc	CBCL Total Problems	Baseline	37.4 ± 12.5	31.0 [27.0–50.0]
ASDc	GSRS	8 weeks	21.0 ± 6.8	19.0 [17.0–22.0]
ASDc	CBCL Internalizing Problems	8 weeks	21.7 ± 5.1	21.0 [18.0–27.0]
ASDc	CBCL Externalizing Problems	8 weeks	12.5 ± 7.8	10.0 [6.0–18.0]
ASDc	CBCL Total Problems	8 weeks	36.6 ± 11.9	31.0 [27.0–48.0]
ASDc	ADOS	8 weeks	6.8 ± 2.4	6.0 [5.0–9.0]
ASDc	CARS	8 weeks	37.4 ± 4.7	38.0 [34.0–41.0]
ASDc	GSRS	6 months	21.0 ± 6.8	19.0 [17.0–22.0]
ASDc	CBCL Internalizing Problems	6 months	21.5 ± 5.4	21.0 [16.0–26.0]
ASDc	CBCL Externalizing Problems	6 months	12.7 ± 7.8	10.0 [7.0–19.0]
ASDc	CBCL Total Problems	6 months	36.7 ± 12.2	30.0 [26.0–50.0]
ASDc	ADOS	6 months	6.9 ± 2.4	6.0 [5.0–10.0]
ASDc	CARS	6 months	37.6 ± 5.4	37.0 [33.0–41.0]
ASDc	PGI-R	8 weeks	0.04 ± 0.06	0.00 [0.0–0.09]
ASDc	PGI-R	6 months	0.07 ± 0.07	0.09 [0.0–0.09]
